# miR-20a Inhibits TCR-Mediated Signaling and Cytokine Production in Human Naïve CD4^+^ T Cells

**DOI:** 10.1371/journal.pone.0125311

**Published:** 2015-04-17

**Authors:** Amarendra V. Reddycherla, Ines Meinert, Annegret Reinhold, Dirk Reinhold, Burkhart Schraven, Luca Simeoni

**Affiliations:** 1 Otto-von-Guericke University, Institute of Molecular and Clinical Immunology, Leipziger Str. 44, Magdeburg, Germany; 2 Department of Immune Control, Helmholtz Centre for Infection Research, Inhoffenstrasse 7, Braunschweig, Germany; University of Iowa, UNITED STATES

## Abstract

Upon TCR stimulation by peptide-MHC complexes, CD4^+^ T cells undergo activation and proliferation. This process will ultimately culminate in T-cell differentiation and the acquisition of effector functions. The production of specific cytokines by differentiated CD4^+^ T cells is crucial for the generation of the appropriate immune response. Altered CD4^+^ T-cell activation and cytokine production result in chronic inflammatory conditions and autoimmune disorders. miRNAs have been shown to be important regulators of T-cell biology. In this study, we have focused our investigation on miR-20a, a member of the miR-17-92 cluster, whose expression is decreased in patients suffering from multiple sclerosis. We have found that miR-20a is rapidly induced upon TCR-triggering in primary human naïve CD4^+^ T cells and that its transcription is regulated in a Erk-, NF-κB-, and Ca^++^-dependent manner. We have further shown that overexpression of miR-20a inhibits TCR-mediated signaling but not the proliferation of primary human naïve CD4^+^ T cells. However, miR-20a overexpression strongly suppresses IL-10 secretion and moderately decreases IL-2, IL-6 and IL8 production, which are crucial regulators of inflammatory responses. Our study suggests that miR-20a is a new player in the regulation of TCR signaling strength and cytokine production.

## Introduction

CD4^+^ T-helper cells play a crucial role in the immune response. Triggering of the T-cell receptor (TCR) by antigens generates a signaling cascade culminating in transcriptional activation, proliferation, differentiation and the generation of a specific T-cell response [1]. An aberrant T-cell activation and differentiation may result in chronic inflammation and autoimmune reactions. Therefore, to prevent severe immunopathological conditions, signaling via the TCR must be tightly regulated.

During the last years, miRNAs have emerged as crucial regulators of T-cell functions and hence have become the target for pharmacological intervention to treat human diseases in which T cells are implicated. miRNAs are a class of small non-coding RNAs of approximately 22 nucleotides in length that function as suppressors of protein expression [2]. They bind to 3’ UTRs of target mRNAs, thus inducing either their degradation or suppressing their translation. miRNAs regulate several aspects of T-cell biology. A recent work has shown that miR-181a, which is highly expressed in thymocytes, can modulate TCR signaling strength and T-cell development by directly targeting certain phosphatases [3]. miRNAs can also modulate the proliferation and differentiation of mature T cells in the periphery. For example, it has been shown that miR-182 can be induced by IL-2 and is involved in clonal expansion of T-helper cells by targeting Foxo1, an inhibitor of cell cycle progression [4]. Additionally, miR-29 set the threshold for Th1/Th2 differentiation [5–7]. miR-155 has been shown to be important for the survival of regulatory T cells (Treg cells) [8]. It has also been shown that miR-326 plays an exclusive role in Th17 differentiation [9], whereas, miR-31 appears to regulate T-cell activation [10].

More recently, the miR-17-92 cluster has been shown to promote and regulate the differentiation of follicular helper T cells (Tfh), a T-cell subset required for B-cell responses and antibody production [11, 12]. This cluster comprises six miRNAs: miR-17, miR-18a, miR-19a, miR-19b, miR-20a, and miR-92a [13]. In addition to the regulation of T-helper cell differentiation, the miR-17-92 cluster is also required for the pro-B to pre-B transition during B-cell development [14]. *In vivo* studies have shown that mice deficient for the miR-17-92 cluster die prematurely [14], whereas transgenic overexpression of the cluster results in severe life threatening lymphoproliferative and autoimmune disorders [15]. In addition to its crucial roles in the immune system, the miR-17-92 cluster has also been shown to regulate cell cycle progression in various cell types by targeting E2F family proteins, P21 and Rbl2 [16–18]. Collectively, these data have demonstrated that the miR-17-92 cluster plays a key role in cell functions. Nevertheless, it is not yet completely understood whether the individual members of this cluster have unique functions. This hypothesis is supported by the following observations. First, intracellular levels of individual miRNAs significantly varied within mouse CD4^+^ T cells [19]. Second, whole blood cells from multiple sclerosis (MS) patients under-express miR-17 and miR-20a, whereas the expression of the other members of the cluster is not affected [20]. This suggests that post-transcriptional regulatory mechanisms allow the selective expression of individual members of this cluster in a particular cellular context. In addition, the functional dissection of the miR-17-92 cluster has also revealed that individual members of the cluster have specific functions. In fact, miR-19b and miR-17 regulate T-cell expansion, Th1 responses, and inhibit iTreg differentiation [19], whereas miR-17 and miR-20a appear to repress the transcription of genes involved in T-cell activation in the Jurkat T-cell line [20].

Here, we have focused our attention on miR-20a, as its expression is rapidly induced (within minutes) upon T-cell activation. When overexpressed in human naïve CD4^+^ T-helper cells, miR-20a inhibits TCR-mediated signaling, CD69 expression, and decreases cytokine production. Collectively, we have demonstrated that miR-20a alone plays a role in the regulation of TCR signaling strength and in cytokine production of CD4^+^ T cells.

## Materials and Methods

### Ethics

Approval for these studies involving the analysis of TCR-mediated signaling in human T cells was obtained from the Ethics Committee of the Medical Faculty at the Otto-von-Guericke University, Magdeburg, Germany with the permission number [107/09]. Informed consent was obtained in writing in accordance with the Declaration of Helsinki.

### Human T-cell purification and culture

Human naïve CD4^+^ T cells were isolated as previously described [21], using human naïve CD4+ T-cell isolation kit (Miltenyi Biotech).

### miRNA expression analysis

Total RNA from approximately 18 nucleotides upwards was isolated using miRNeasy kit (Quiagen), according to the manufacturer’s instructions. Equal amounts of total RNA from each sample was used for reverse transcription of miRNAs using the miScript II RT kit supplied by Qiagen. Real-time PCR analysis was done by using miScript SYBR Green PCR kit (Qiagen) and the ABI Prism 7000 Sequence Detection System (Applied Biosystem). 3 ng (for mature miRNA) and 15 ng (for precursor miRNAs) from each sample were used for the Real-time PCR analysis. The small RNA, RNU6B was used as reference gene and the primers for the detection of mature miRNAs, miRNA precursors (both pri- and pre-miRNAs), and the reference gene (RNU6B) were purchased from Qiagen (miScript Primer Assays). Differences in gene expression were calculated by the Δ Δ Ct method and the data were normalized to the reference gene RNU6B. The quality and quantity of RNA and cDNA was evaluated by measuring the OD at 260 and 280 nm with Ultrospec 3000 (Pharmacia Biotech).

To investigate transcriptional regulation of miR-20a, 1x10^6^ cells were pre-incubated with U0126 (10 μM; Cell Signaling), IKK VII (200 nM; Calbiochem), or EGTA (10 mM; Sigma-Aldrich) for 30 min at 37°C and were stimulated with plate-bound antibodies as described above. DMSO (Sigma-Aldrich) was used as negative control. Expression of miR-20a precursors was investigated as described above.

### Overexpression of miR-20a

For miR-20a overexpression studies, a BLOCK-iT Pol II miR RNAi Expression Vector Kit (Invitrogen) was used to generate a vector coding for either miR-20a or a miR-control. The pre-miR-20a sequence was derived from the miRBase database (http://microrna.sanger.ac.uk/) and was chemically synthesized (Biomers.net). The pre-miR-20a sequence additionally includes overhangs for cloning into the pcDNA 6.2-GW/EmGFP-miR vector (Invitrogen). The following sequences were designed: Top strand: 5’-GTAGCACTAAAGTGCTTA TAGTGCAAGTAGTGTTTAGTTATCTACT GCATTATGAGCACTTAAAGTACTGC-3’.

Bottom strand: 3’–GCAGTACTTTAAGTGCTCATAATGCAGTAGATAACTAAACAC TA CCTGCACTATAAGCACTTTAGTGCTA-5’. A negative control plasmid was included in the BLOCK-iT Pol II miR RNAi Expression Vector Kit (Invitrogen). For overexpression, 1 x 10^6^ human naïve CD4^+^ T cells were transfected with 1 μg of either miR-control or miR-20a expressing plasmids using AMAXA nucleofection kit (Lonza) according to the manufacturer’s instructions. After transfection T-cells were transferred to 6-well culture plates containing RPMI 1640 medium (Biochrome) supplemented with 10% FCS and 2 μg/mL Ciprobay and incubated for 16 h before use.

### Suppression of miR-20a

To suppress miR-20a, DNA sequences complementary to the miR-20a (Decoy) were chemically synthesized from biomers.net. The following decoys were used; miR-20a Decoy: 5’-CTAAACACTACCTGCACTATAAGCACTTTAGTGCTAC-3’; Scramble decoy: 5’-GAAATGTACTGCGCGTGGAGACGTTTTGGCCACTGAC-3’. Briefly, human naïve CD4^+^ T cells were washed with PBS and resuspended in OPTI-MEM I (Gibco). 1 x 10^6^ human naïve CD4^+^ T cells were transfected with 1 μM of either miR-20a decoy or scramble control using Genepulser Xcell (BIO-RAD), rested for 16h in 6-well culture plates containing RPMI 1640 medium (Biochrome) supplemented with 10% FCS and 2 μg/mL Ciprobay before use.

### Western blotting

For Western blot analyses, T cells were stimulated with antibody-coated microbeads and cell lysates were prepared and assayed as previously described [21, 22]. The following antibodies were used for Western blotting: anti-pY^319^Zap-70, anti-pY^171^LAT, anti-pY^738^PLC-γ, anti-pT^202^/Y^204^Erk1/2, STAT3, BIM, PTEN (all from Cell Signaling), Total Lck, PLC-γ, SLP-76, SOS (all from Santa Cruz), ZAP-70, LAT, GADS (Upastate), Erk (Promega) and anti-β-actin (Sigma-Aldrich). Quanitification of the Western blots was performed as previously described [22]. Briefly, densities of detected bands on blots were acquired using Epson Perfection V700 Photo Scanner (Epson) and the net intensity of bands was quantified by using ImageQuant software (Kodak) and the values were normalized to corresponding β-actin.

### Immunoprecipitation

Primary human naïve CD4^+^ T cells (3×10^7^) were either left untreated or stimulated with iAbs for the indicated periods of time. Cells were lysed in 1% Brj58 or 1% lauryl maltoside (N-dodecyl β-maltoside), 1% IGEPAL CA-630, 1 mM Na_3_VO_4_, 1 mM PMSF, 10 mM NaF, 10 mM EDTA, 50 mM Tris pH 7.5, and 150 mM NaCl, and cleared by centrifugation. TCRζ chains were then immunoprecipitated with agarose-conjugated CD3ζ (Santa Cruz Biotechnology) antibody followed by recombinant protein A-agarose beads (Santa Cruz Biotechnology) at 4°C overnight. After washing, TCRζ immunoprecipitates were resolved by SDS-PAGE, transferred to a nitrocellulose membrane (Amersham), and analyzed by immunoblotting with the indicated antibody.

### Ca^++^ measurement

To measure Ca^++^ flux, cells were transfected with miRNA vectors as described above and incubated with 4 μM Indo-1 AM (Invitrogen, Molecular Probes) at 37°C for 45 min. Cells were then washed, resuspended in Hank’s buffer (Biochrome), and stimulated with soluble CD3ε (clone MEM 92, kindly provided by V. Horejsi, Academy of Sciences of the Czech Republic, Czech Republic) and CD28 (clone 248.23.2; [23]) mAbs. Ca^++^ flux was measured on a LSRII flow cytometer (BD Biosciences). Ionomycin was added to the samples at the end of the measurement as a positive control to measure maximum Ca^++^ flux. Raw data were transferred to FlowJo V3.6.1 (Tree Star, USA) for the analysis.

### Functional assays

Human naïve CD4^+^ T cells were isolated and transfected with miRNA vectors as described above. 16 hours after transfection, 1x10^6^ cells were seeded onto 48-well plates that were coated with goat anti-mouse IgG + IgM (H+L) (Dianova) at a concentration of 4.5 μg/mL and with mouse-anti-human CD3ε (clone MEM 92, kindly provided by V. Horejsi, Academy of Sciences of the Czech Republic, Czech Republic) or mouse-anti-human CD28 hybridoma supernatents (clone 248.23.2; [23]). For CD69 expression T cells were cultured for 5 h. Subsequently, cells were stained with APC-conjuagated CD69 mAbs (BD Bioscience) at 1:10 dilution and incubated at 4°C for 15 min. T cells were then washed with cold PBS and the surface expression of CD69 was measured by flow cytometry (LSRFortessa, BD Biosciences). Analysis was done with FlowJo V3.6.1 (Tree Star, USA). For cytokine measurements, T cells were cultured for 48 h in X-VIVO 15 serum free medium (Lonza). Subsequently, supernatants were collected and cytokine concentrations were measured by ELISA (R&D Systems) or by Multiplex Cytokine Assay (Bio-Rad) according to manufacturer’s instructions.

For proliferation, transfected human naïve CD4^+^ T cells were labeled with 5 μM Alexa Fluor 700 (eBioscience) for 10 min at 37°C. After washing, 1.5×10^6^ cells were seeded in 24 well plate and cultured in 1000 μL RPMI (supplemented with 10% FCS and antibiotics). Cells were either left unstimulated or stimulated with plate bound antibodies as described above and were cultured for 60 h at 37°C, 5% CO_2_. Proliferation was assessed by Alexa Fluor 700 dye dilution by flow cytometry by gating on GFP^+^ cells using a BD LSRFortessa, FACSDiva Software 6.1.3 (BD Biosciences), and FlowJo V3.6.1 (Tree Star, Inc.).

### Statistical analysis

Statistical analysis was performed using GraphPad Prism 5. *P* values were determined by two tailed Student’s *t* Test. *P* values < 0.05 were considered as significant.

## Results

### miR-20a is upregulated upon T-cell activation

**Fig 1:**
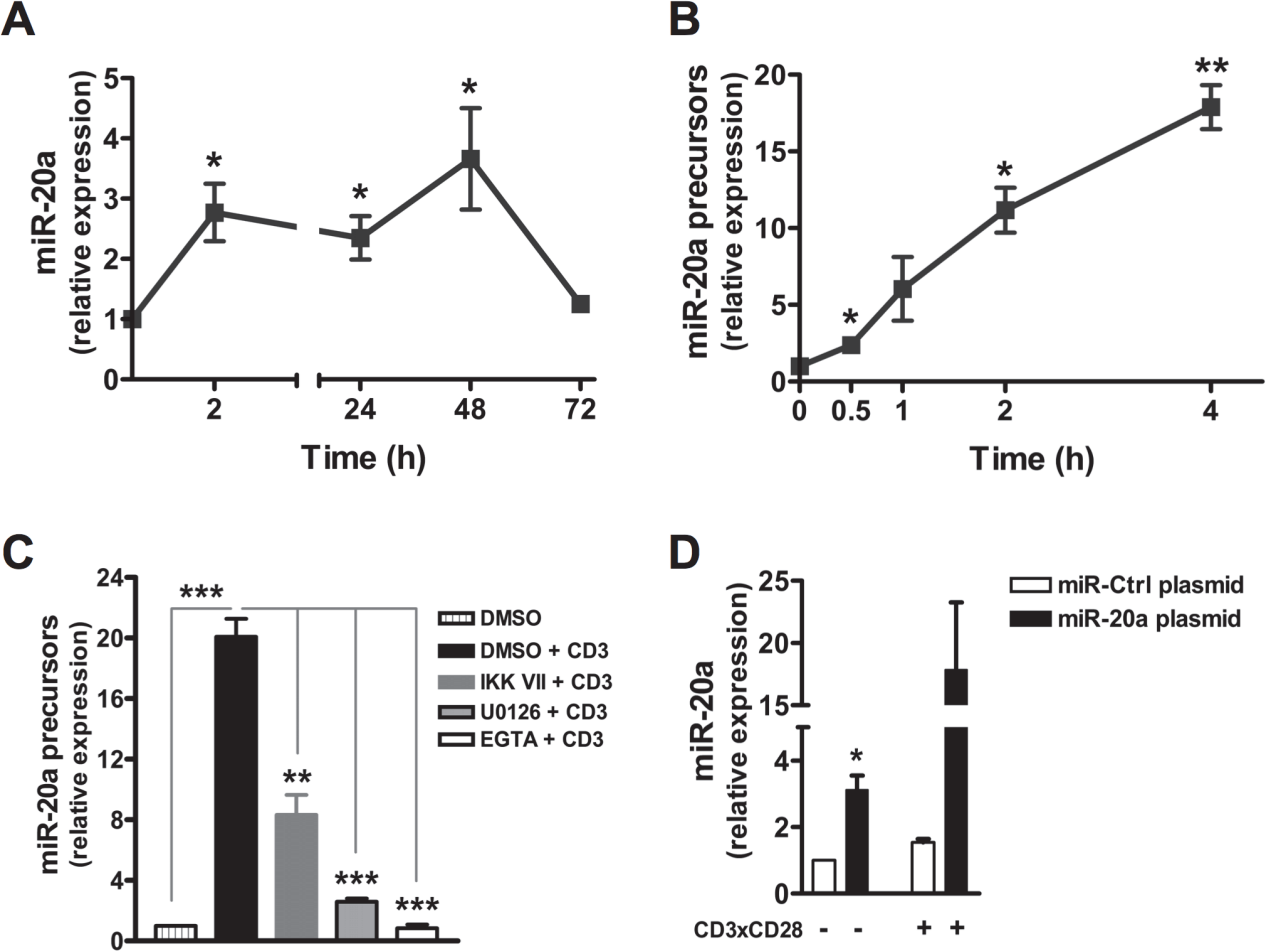
Expression of miR-20a and miR-20a precursors in human naïve CD4^+^ T cells. Human naïve CD4^+^ T cells were stimulated with plate-bound CD3 and CD28 mAbs for the indicated time periods. Quantification of **(A)** miR-20a and **(B)** miR-20a precursors (both pri- and pre-miRNAs) was performed using RT qPCR. **(C)** Naïve CD4^+^ T cells were also preincubated with DMSO, U0126 (10 μM), IKK VII (200 nM) and EGTA (10 mM) for 30 minutes. Subsequently, samples were either left unstimulated or stimulated with plate-bound CD3 for 4 hours at 37°C. Expression of miR-20a precursors was quantified using RT qPCR. **(D)** Quantification of miR-20a level upon overexpression. Human naïve CD4^+^ T cells were transfected with plasmids encoding either miR-20a or miR-control (miR-Ctrl). Cells were either left unstimulated or stimulated for 2 hours with CD3xCD28 mAbs. Quantification of miR-20a in both resting and activated cells was performed using real-time PCR. Unless otherwise mentioned, all the data was normalized to either resting cells or unstimulated control transfected cells. Data represent arbitrary units ± SEM of at least 3 (A, C, D) and 2 (B) independent experiments. Significant P values were done by using Student’s t Test. (*, P < 0.05; **, P < 0.01; ***, P < 0.001).

To investigate whether miR-20a is involved in human naïve CD4^+^ T-cell activation, we first determined the expression pattern of miR-20a upon TCR triggering. Freshly purified naïve CD4^+^ T cells from healthy donors were stimulated with plate-bound CD3 and CD28 monoclonal antibodies (mAbs). As shown in Fig 1A, the expression of miR-20a was rapidly (within 2 h) and significantly upregulated after T-cell stimulation. In addition to a rapid induction, the expression of miR-20a was also sustained up to 48 h and decreased thereafter to the basal level (Fig 1A).

Mature miRNAs are processed from miRNAs precursors, which are the result of miRNA gene transcription [24]. To investigate whether miRNA-20a is *de novo* transcribed upon TCR stimulation, we analyzed the expression of miRNA-20a precursors (both pri- and pre-miRNAs). Real-time quantitative PCR (RT qPCR) analysis showed that the expression of miR-20a precursors (both pri- and pre-miRNAs) was induced very rapidly (detectable already after 30 min) upon TCR triggering (Fig 1B). These data indicate that miR-20a is rapidly synthesized upon T-cell activation and suggest that it may play a role in early activation events.

### TCR-mediated transactivation of miR-20a depends on Erk, Ca^++^ and NF-κB

Signals transduced via the TCR result in the activation of AP1, NF-κB and NFAT, three key transcription factors required for the induction of crucial genes (e.g. IL-2) and hence for T-cell proliferation. Activation of AP1, NF-κB and NFAT depends on the activation of Erk, IKK and on Ca^++^ flux, respectively. To investigate whether these signaling pathways are also involved in the transactivation of miR-20a in response to TCR stimulation, we measured the levels of miR-20a precursors in activated CD4^+^ T-cells in the presence or absence of U0126, IKK VII, and EGTA which selectively inhibit the activation of Erk, NF-κB, and inhibit Ca^++^ flux, respectively. As shown in Fig 1C, transcription of miR-20a was inhibited about 50% and 85% upon treatment with IKK VII and U0126, respectively, compared to control cells treated with DMSO alone. Interestingly, inhibition of Ca^++^ flux by EGTA completely abolished the expression of miR-20a precursors. Collectively, these results suggest that miR-20a transcription strongly depends on Ca^++^ flux, and Erk signaling and to a lesser extent on NF-κB activity.

### miR-20a negatively regulates TCR-mediated signaling

Having shown that miR-20a is rapidly induced upon TCR stimulation and that its expression depends on transcription factors which are crucial for T-cell functions, we next assessed whether miR-20a is involved in the regulation of T-cell activation. We performed overexpression studies using human naïve CD4^+^ T cells. In order to overexpress miR-20a, we cloned the human pre-miR-20a sequence into a pcDNA6.2-GW/EmGFP miR expression vector. A plasmid coding for a miR-scrambled sequence, which does not target any known vertebrate gene, was used as control. Using this system we were able to achieve a moderate overexpression (about 3 folds, Fig 1D) of miR-20a in resting CD4^+^ T cells with about 70% transfection efficiency (data not shown). The overexpression levels were comparable to the physiological levels found in activated human primary naïve CD4^+^ T cells (Fig 1A). Efficient overexpression of miR-20a was also observed upon TCR stimulation of resting CD4^+^ T cells (Fig 1D).

**Fig 2:**
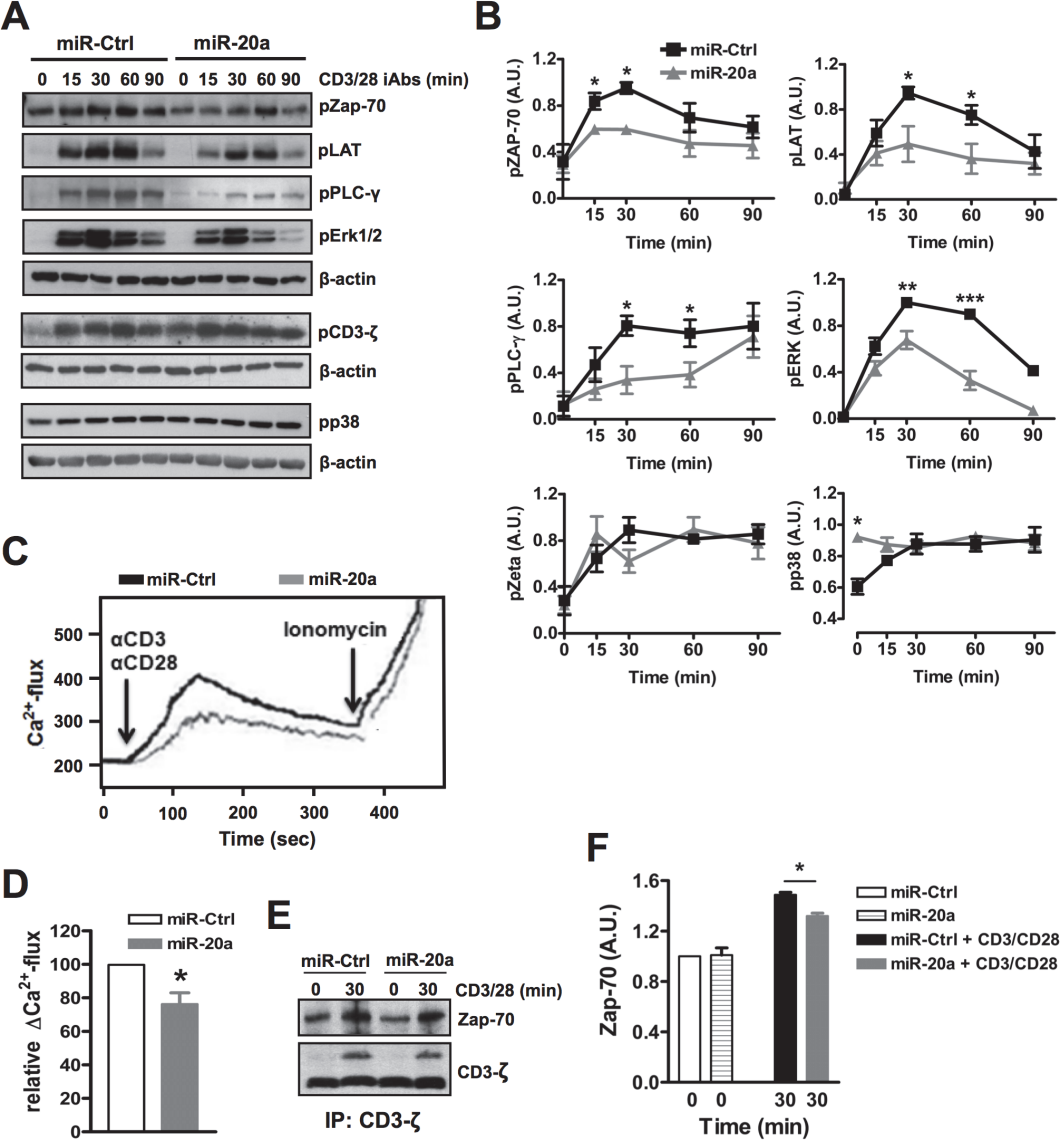
miR-20a inhibits TCR-mediated signaling. Human naïve CD4^+^ T cells were transfected with plasmids encoding either miR-20a or miR-control (miR-Ctrl) and cultered for 16 hours. **(A)** Cells were stimulated with microbeads coated with CD3 and CD28 mAbs (iAbs) for the indicated time periods. Subsequently, lysates were analyzed by immunoblotting using the indicated Abs. Bands in **(A)** were quantified using the ImageQuant software and values were normalized to the corresponding β-actin signal. Graphs in **(B)** show the phosphorylation levels of the indicated molecules as arbitrary units ± SEM of at least 4 independent experiments. **(C)** CD4^+^ T cells were incubated with Indo-1AM, stimulated with CD3 and CD28 mAbs, and Ca^++^ flux was measured by flow cytometry. Ionomycin is used to induce maximum Ca^++^ flux. Graph in **(D)** shows quantification of Ca^++^ flux expressed as arbitrary units ± SEM of at least 3 independent experiments. **(E)** CD4^+^ T cells were stimulated with iAbs for the indicated time periods. Subsequently, cell lysates were prepared and CD3ζ immunoprecipitations were analyzed by immunoblotting using the indicated Abs. Bands in **(E)** were quantified as described above. Graph in **(F)** shows the levels of CD3ζ-associated Zap-70 as arbitrary units ± SEM of 2 independent experiments. Significat *P* values were done by using Student’s *t* Test. (*, *P* < 0.05; **, *P* < 0.01; ***, *P* < 0.001).

Following TCR stimulation, two key tyrosine kinases, Lck and Zap-70, initiate signaling leading to gene transcription and T-cell responses [25]. Lck and Zap-70 activation is required for the formation of a signaling complex organized by the transmembrane adaptor protein LAT, which in turn will orchestrate the activation of PLC-γ1. PLC-γ1 generates second messengers mediating Ras-Erk1/2 signaling, PKCθ-CBM, and Ca^++^-Calcineurin pathways, which result in turn in the activation of AP-1, NF-κB, and NFAT, respectively. To investigate whether miR-20a regulates T-cell activation, we initially performed a biochemical analysis of TCR signaling. Freshly purified naïve CD4^+^ T cells were transfected with plasmid encoding either miR-20a or miR-control. Subsequently, cells were stimulated with CD3 and CD28 mAbs immobilized on microbeads (iAbs). As shown in Fig 2A and 2B, overexpression of miR-20a inhibits the TCR-mediated phosphorylation of Zap-70, LAT, PLC-γ, and Erk1/2. In addition, overexpression of miR-20a results in the constitutive activation of p38 mitogen- activated protein kinase (Fig 2A and 2B). Moreover, it appears that p38 phosphorylation cannot be further induced upon TCR stimulation in cells overexpressing miR-20a. Given that activated PLCγ-1 mediates Ca^++^ signaling by generating IP_3_, a decrease in the phosphorylation of PLCγ-1 upon miR-20a overexpression should also result in the reduction of the release of intracellular Ca^++^ in response to TCR stimulation. As expected, we have found that miR-20a overexpressing cells showed indeed a reduction in Ca^++^ flux compared to cells expressing miR-negative control (Fig 2C and 2D). We next assessed whether, miR-20a affects the activation of signaling molecules upstream of Zap-70. However, we did not observe any decrease in the phosphorylation of CD3ζ (Fig 2A and 2B). To test whether the inhibition of TCR-mediated signaling is due to a reduction in the recruitment of Zap-70 to CD3ζ, we performed CD3ζ immunoprecipitations. As shown in Fig 2E and 2F, we observed a slight dicrease in the recruitment of ZAP70 to CD3ζ upon miR-20a overexpression compared to miR-control. Collectively, these data indicate that miR-20a regulates TCR-mediated signaling at the level of Zap-70 activation.

**Fig 3:**
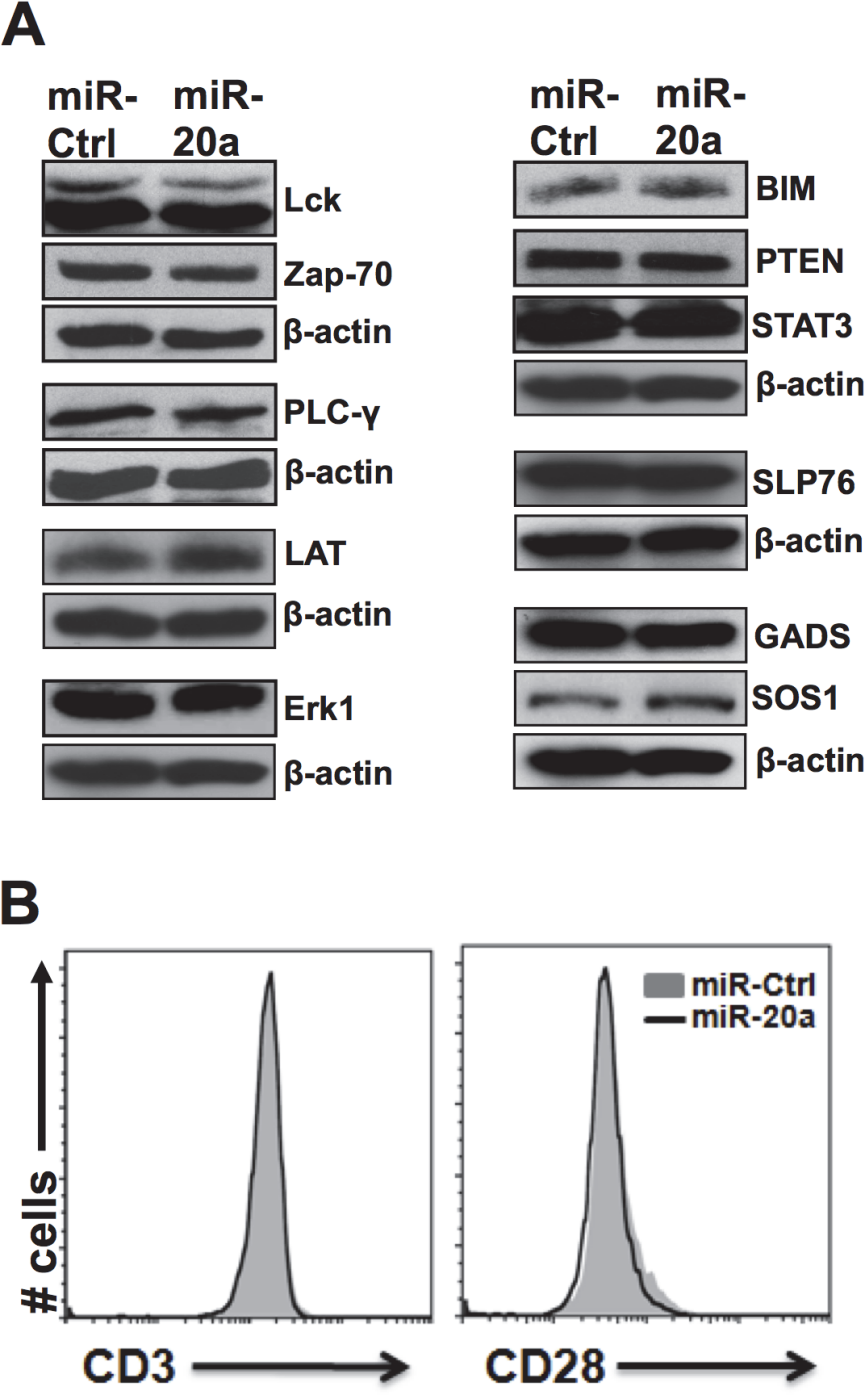
miR-20a does not affect the expression of signaling molecules and CD3 and CD28 receptors. **(A)** Lysates from T cells transfected with either miR-20a or miR-control were prepared and analyzed by immunoblotting using the indicated Abs. (B) CD4^+^ T cells overexpressing either miR-20a or miR-control cells were stained with antibodies against CD3 and CD28 and analyzed by flow cytometry. Histograms overlay show the expression of CD3 (left) and CD28 (right) in miR-20a versus miR-control transfected cells.

To exclude the possibility that the overexpression of miR-20a affects the expression of crucial signaling molecules, we performed additional experiments. We have found that overexpression of miR-20a does not affect the expression of Lck, Zap-70, LAT, PLC-γ, SLP-76, GADS, Sos1, Erk in resting (Fig 3A) as well as in activated CD4^+^ T cells (data not shown). Furthermore, there is also no difference observed in the expression levels of BIM, PTEN and STAT3 that have been previously shown to be the targets of the miR-17-92 cluster in both resting (Fig 3A) and activated CD4^+^ T cells (data not shown). Moreover, to exclude the possibility that defective TCR signaling is due to reduced receptor levels, we also examined the expression of CD3 and CD28. FACS analyses revealed that miR-20a overexpression does not change the densities of CD3 and CD28 on the cell surface compared to control (Fig 3B).

**Fig 4:**
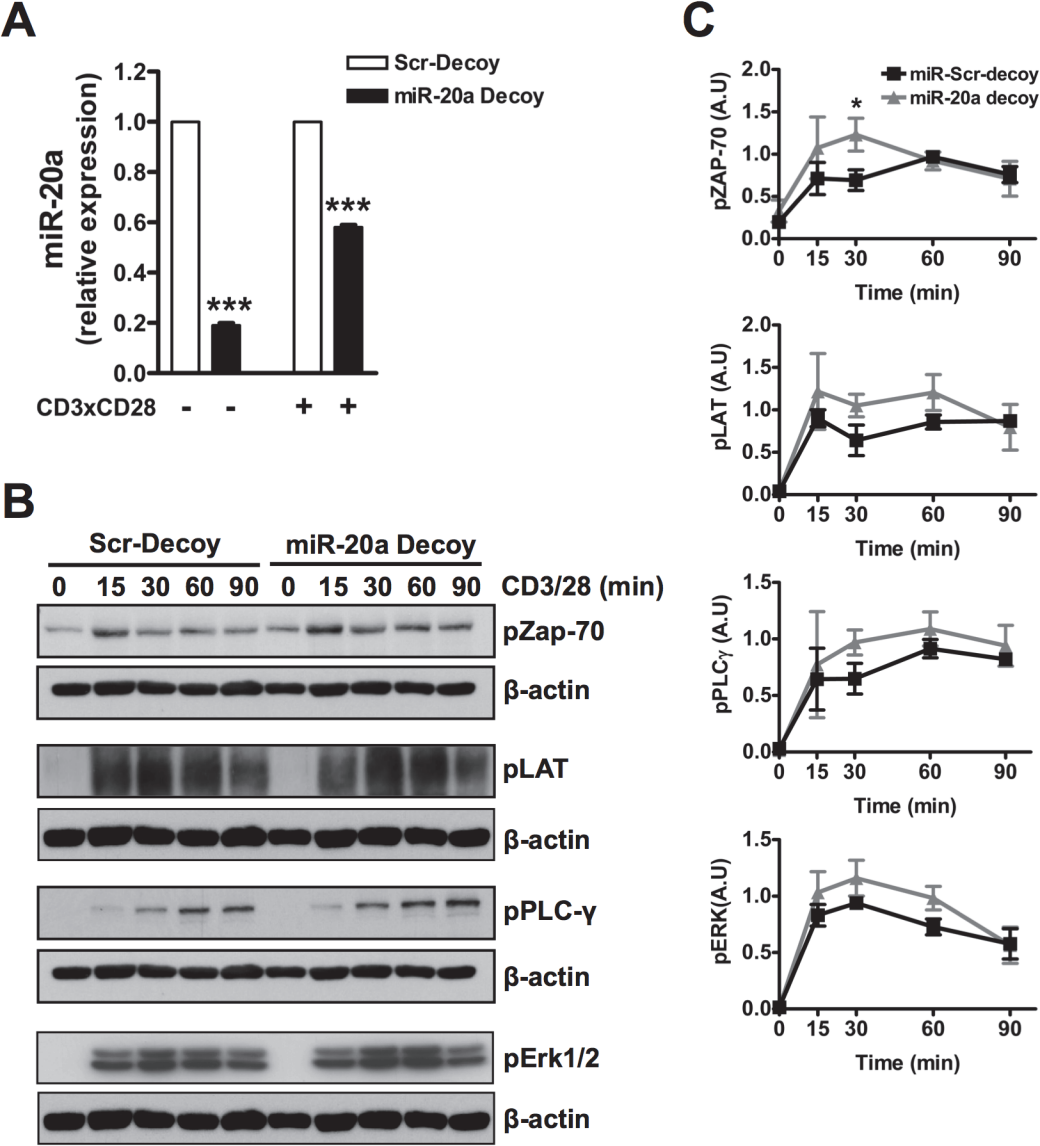
Inhibition of miR-20a slightly increases TCR-mediated signaling. Human naïve CD4^+^ T cells were transfected with miR-20a or scramble decoys. **(A)** Quantification of miR-20a expression in resting and in stimulated cells upon stimulation with plate-bound CD3 and CD28 mAbs for 45 min was performed using RT qPCR. **(B)** 16 hours after transfection, cells were stimulated with microbeads coated with CD3 and CD28 mAbs for the indicated time periods. Subsequently, lysates were analyzed by immunoblotting using the indicated Abs. Bands in **(B)** were quantified using the ImageQuant software and values were normalized to the corresponding β-actin signal. Graphs in **(C)** show the phosphorylation levels of the indicated molecules as arbitrary units ± SEM of 3 independent experiments. Significat P values were calculated by using Student’s t Test. (*, P < 0.05).

In order to shed more light onto the function of miR-20a in TCR-mediated signaling, we performed inhibition experiments using a miR-20a decoy. As shown in Fig 4A, we were able to achieve an efficient suppression (about 80%) of miR-20a in resting CD4^+^ naïve T cells. However, 45 minutes after TCR stimulation inhibition was less efficient, as decoy-treated CD4^+^ T cells displayed only 35% reduction of miR-20a expression compared to scrambled controls (Fig 4A). When we assessed TCR-mediated signaling, we observed an increase in the phosphorylation of different signaling molecules upon treatment with miR-20a decoy (Fig 4B and 4C). These data are in agreement with the overexpression data and support the hypothesis that miR-20a is a negative regulator of TCR-mediated signaling. However, the difference between decoy- and scrambled-treated T cells is only minor. Based upon these data, we used the overexpression system for further analyses.

### Reduced CD69 expression but normal T-cell proliferation upon miR-20a overexpression

**Fig 5:**
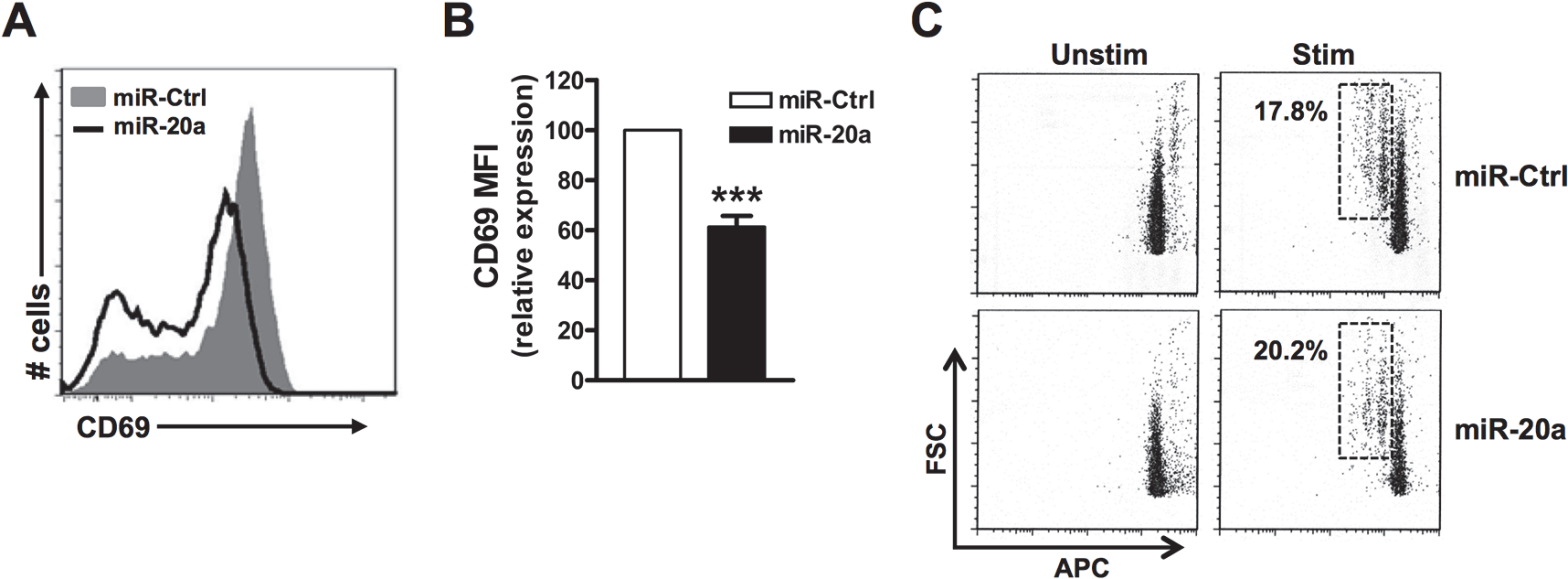
miR-20a inhibits early T-cell activation events. Human naïve CD4^+^ T cells were transfected with either miR-20a or miR- control expression plasmids. **(A)** 16h after transfection, cells were stimulated with immobilized CD3xCD28 mAbs for 6 hours and stained with an APC-conjugated CD69 mAb. Surface expression of CD69 was measured by flow cytometry. One representative experiment of 4 is shown. Graph in (**B**) represents relative mean fluorescent intensity (MFI) of CD69 expression of miR-20a overexpressing cells compared to miR-control. **(C)** Alexa Fluor 700 labelled cells were either left unstimulated or stimulated with platebound CD3xCD28 antibodies for 60 hours. Proliferation of cells was then analysed by flow cytometry by measuring the dilution of the fluorochrome by gating on GFP+ cells. Data represent arbitrary units ± SEM of at least 3 independent experiments. Significat *P* values were done by using Student’s *t* Test. (***, *P* < 0.001).

TCR triggering result in a rapid transcription of the CD69 gene, an early T-cell activation marker whose expression depends on the Ras-Erk cascade [26, 27]. As TCR-mediated signaling is defective upon overexpression of miR-20a, we next tested whether miR-20a also affects T-cell activation at a transcriptional level and we measured CD69 expression as a read-out system. As shown in Fig 5A and 5B, overexpression of miR-20a significantly decreased CD69 expression compared to cells transfected with miR-control.

At later stages, T-cell activation results also in CD25 expression and IL-2 production, which lead to proliferation and expansion of T cells. We next assessed whether these processes are affected upon overexpression of miR-20a. We have found that CD25 is upregulated to similar levels in both control and miR-20a overexpressing cells (data not shown). Finally, also CD4^+^ T-cell proliferation was not affected upon miR-20a overexpression (Fig 5C). Thus, these data corroborate the idea that miR-20a is involved in the regulation of early transcriptional events during the activation of T cells [20], but it appears to be dispensable for T-cell proliferation.

### miR-20a inhibits cytokine production

**Fig 6:**
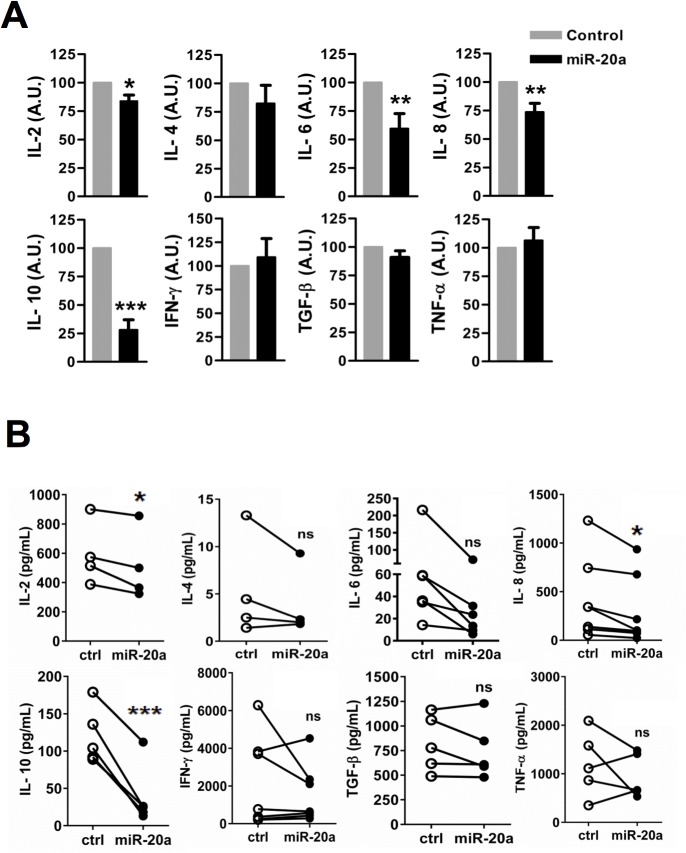
miR-20a regulates cytokine production. Human naïve CD4^+^ T cells were transfected with plasmids encoding either miR-20a or miR-control (miR-Ctrl). 16 h after transfection, cells were stimulated with plate-bound CD3 and CD28. 48 h after stimulation, supernatants were collected and cytokines were measured by ELISA. Graphs in **(A)** show relative cytokine levels from miR-20a overexpressing cells compared to miR-control. Graphs in **(B)** show the absolute values of cytokine concentration, in pg/mL, from individual experiments. Data expressed as arbitrary units ± SEM of at least 4 independent experiments. Significat *P* values were done by using Student’s *t* Test. (*, *P* < 0.05; **, *P* < 0.01; ***, *P* < 0.001).

We next investigated whether miR-20a is involved in the regulation of cytokine production. We measured cytokines such as IL-2, IL-4, IL-6, IL-8, IL-10, IFN-γ, TGF-β and TNF-α. As shown in Fig 6A, overexpression of miR-20a moderately decreased the production of IL-2, IL-6, IL-8, but strongly inhibited IL-10 secretion compared to miR-control. On the other hand, overexpression of miR-20a did not affect the release of other cytokines such as IL-4, IFN-γ, TGF-β and TNF-α. Absolute cytokine concentrations are provided in Fig 6B. Collectively, our data indicate that miR-20a play an important role in cytokine production in CD4^+^ T cells.

## Discussion

CD4^+^ T cells are not only key regulators of immune responses but are also involved in pathological processes such as allergy, chronic inflammation and autoimmunity. During the last years, it has been shown that miRNAs are involved in the regulation of T-cell development and differentiation [28]. Recently, the miR-17-92 cluster has emerged as a pivotal regulator of immune cells. Transgenic overexpression of this cluster in mice leads to spontaneous activation of the lymphocyte compartment associated with severe lymphoproliferative disorders [15]. In agreement with these observations, the miR-17-92 cluster is frequently overexpressed in hematopoietic malignancies [29]. It has been additionally shown that its deletion leads to defects in B-cell development and increased B-cell apoptosis [14]. Interestingly, miR-17-92 cluster regulates differentiation of helper CD4^+^ T cells. In particular, it promotes Tfh responses [11, 12] and is required for the fitness of regulatory T cells [30]. In CD8^+^ T cells, this cluster regulates effector and memory CD8^+^ T-cell differentiation by targeting multiple negative regulators of the PI3K-Akt-mTOR axis, such as PTEN, PD1 and BTLA [31]. Recently, the function of the individual members of this cluster has also become focus of research. It has been shown that miR-17 and miR-19b regulate Th1 and prevents inducible Treg differentiation by targeting PTEN, TGFβRII, and CREB1 [19], and also promote Th17-mediated inflammation by targeting PTEN and IKZF4 [32] in mouse CD4^+^ T cells.

Here, we have selectively investigated the function of miR-20a in human naïve CD4^+^ T cells. In fact, it has been shown that miR-20a represses transcriptional activation in the Jurkat T-cell line and was found to be decreased in multiple sclerosis patients’ whole blood samples [20]. We have found that miR-20a is rapidly induced upon stimulation of the TCR and that its expression depends on the three key signaling pathways Ca^++^, Erk, and NF-κB. Upon overexpression, miR-20a negatively regulates TCR-mediated signaling by inhibiting the phosphorylation of ZAP70, LAT, PLC-γ and Erk and also CD69 expression. These data corroborate previous observations suggesting that miR-17 and miR-20a repress transcriptional activation in the Jurkat T-cell line [20]. To confirm the role of miR-20a in T cell activation, we suppressed its expression by DNA decoys. Suppression of miR-20a moderately increased the phosphorylation of different signaling molecules upon TCR stimulation. These results are in agreement with the overexpression data and support the hypothesis that miR-20a is a negative regulator of TCR-mediated signaling. Nevertheless, the observed differences between decoy- and scrambled-treated CD4^+^ T cells are only minor. We propose that this could either be due to the rapid re-expression of miR-20a upon TCR stimulation or to redundancy among members of the miR17-92 cluster and its paralogs (miR-106a-363 and miR-106b-25). Indeed, it has been shown that miR-20a functionally overlaps with miR-17, miR-106a, or miR-106b in macrophages, myeloid, and neuronal cells [33–36]. Additionally, the seed region, which is crucial for the recognition of the mRNA target, is highly conserved among miR-17, miR-20a, miR-20b, miR-93, miR-106a, and miR-106b [13].

Despite defective TCR-mediated signaling and CD69 expression, overexpression of miR-20a did not affect T-cell proliferation. The latter observation is in line with normal upregulation of CD25 and the almost comparable levels of IL-2 production in miR-20a overexpressing vs. control cells. Moreover, these observations also corroborate previous data showing that miR-19b, but not miR-20a, regulates mouse CD4^+^ T-cell proliferation upon antigen stimulation [19]. We additionally investigated whether miR-20a is involved in cytokine production. We have found that miR-20a inhibits the secretion of cytokines such as IL-6, IL-8 and IL-10. Therefore, we favor the hypothesis that miR-20a is not involved in the regulation of T-cell proliferation but rather in the cytokine production in CD4^+^ T cells. A number of studies have demonstrated that TCR-dependent signal strength regulates T-cell polarization [37–39]. The regulation of the magnitude or the duration of signaling is used in many biological systems to control cell fate decisions [40]. Thus, we hypothesize that miR-20a may be a part of the cell machinery that translates quantitative differences in TCR signaling into qualitative regulation of CD4^+^ T-cell cytokine secretion.

Under non-polarizing conditions, we observed that miR-20a moderately decreased the production of IL-8, which is known as chemotactic factor [41,42]. Therefore, we suspect that miR-20a may limit the recruitment of inflammatory cells at the site of inflammation. Interestingly, a previous study showed that IL-8 is a target of miR-17 and miR-20a in breast cancer cell lines [43]. Thus, IL-8, which has been shown to promote pro-inflammatory response, is a target of miR-20a not only in T cells.

Also IL-6 production was suppressed upon miR-20a overexpression. Upon infection, IL-6 production strongly contributes to host defense. However, elevated levels of IL-6 are associated with the development of chronic autoimmune and inflammatory diseases such as rheumatoid arthritis, Crohn´s disease, and Castleman´s disease. Indeed, the blockade of the IL-6/IL-6R signaling axis using tocilizumab, a humanized anti-IL-6R antibody, has become a therapeutic option to treat autoimmune diseases [44, 45]. IL-6 is produced not only by innate immune cells but also by CD4^+^ effector helper T cells. Several effects of IL-6 on CD4^+^ T cells have been described including the promotion of cell survival, differentiation of Th2 and Th17 cells, the inhibition of Th1 differentiation, and the stimulation of antibody production by B cells [46].

In contrast to the mild effect on IL-2, IL-6, and IL-8 secretion, miR-20a appears to strongly suppress IL-10 production in human naïve CD4^+^ T cells. It is important to mention that IL-10 concentration is also decreased upon ectopic expression of miR-17 and miR-20a in breast cancer cell lines [43]. IL-10 possesses anti-inflammatory properties [47–49]. IL-10-deficient mice exhibit prolonged inflammatory responses under a variety of experimental conditions, whereas in humans the levels of IL-10 inversely correlate with the severity of autoimmune diseases. Different immune cells release IL-10 including macrophages, B cells, CD8^+^ and CD4^+^ T cells. IL-10 enhances the suppressiveness of Treg subsets, attenuates the pathogenicity of Th17 cells, and inhibits both Th1 and Th2 responses [48].

Thus, our data suggest that miR-20a alone is a regulator of cytokine production in naïve CD4^+^ human T cells and hence may play an important role in the homeostasis of the adaptive immune system. How miR-20a regulates interleukin production is not yet clear. It has been shown that the TCR-mediated production of IL-2 and IL-6 depends on Erk1/2 and Ca^++^ signaling [50]. Similarly, IL-10 secretion in CD4^+^ T cells also depends on TCR-mediated Erk1/2 activity [51]. In addition, it has also been shown that p38 mediates the expression of IL-10 in T cells [52, 53]. Based on the observation that Erk1/2, and Ca^++^, and p38 signaling are all altered upon miR-20a overexpression, we hypothesize that miR-20a may regulate cytokine production at least in part via TCR-mediated signaling. Nevertheless, we do not exclude the possibility that miR-20a may also regulate cytokine production via mechanisms which are independent on TCR signaling, for example by directly targeting cytokine transcripts.

One of the important findings of our work is that miR-20a inhibits TCR signaling at a very proximal level. PTEN has been shown as a validated target of miR-17-92 cluster [15]. PTEN is a crucial regulator of proximal signaling also in T cells [54] and hence it could represent an ideal target candidate for the regulation of TCR signaling mediated by miR-20a. However we did not observe any change in PTEN upon miR-20a overexpression in T cells, which appears rather to be a target of miR-17 and miR-19b, as suggested by recent data in mouse CD4^+^ T cells [19, 32]. STAT3 has been shown to be a target of miR-20a in HEK 293 and RAW 264.7 cell lines [55]. Similarly to PTEN, we also did not find alterations in STAT3 expression upon miR-20a overexpression.

Interestingly, a recent study has demonstrated that the miR-17-92 cluster regulates B-cell receptor signaling [56]. The data suggest that this cluster targets CD22 and FCGR2B, two immunoreceptor tyrosine inhibitory motif (ITIM)-containing proteins. miR-17-92 expression is required for the phosphorylation of Syk and BLNK and Ca^++^ flux upon BCR stimulation. Therefore, it is also plausible that this cluster regulates TCR signaling. Collectively, it appears that members of the miR-17-92 cluster have different targets and diverse functions in different cell types and may be even in different species.

In conclusion, we have shown for the first time that miR-20a negatively regulates TCR-mediated signaling and cytokine production. Thus, miR-20a represents a novel potential target for the modulation of inflammatory conditions, for the treatment of autoimmune diseases, and for the regulation of antibody responses. Further studies using *in vivo* models and the identification of the molecular targets of miR-20a are required to shed more light onto the physiological function of miR-20a and onto the mechanism of its action.

## References

[1] Weiss A, Littman DR. Signal transduction by lymphocyte antigen receptors. Cell. 1994; 76: 263–274. 8293463 10.1016/0092-8674(94)90334-4

[2] Ambros V. The functions of animal microRNAs. Nature. 2004; 431: 350–355. 15372042 10.1038/nature02871

[3] Li Q-J, Chau J, Ebert PJR, Sylvester G, Min H, Liu G, Braich R, et al miR-181a is an intrinsic modulator of T cell sensitivity and selection. Cell. 2007; 129: 147–161. 17382377 10.1016/j.cell.2007.03.008

[4] Stittrich A-B, Haftmann C, Sgouroudis E, Kühl AA, Hegazy AN, Panse I, Riedel R, et al The microRNA miR-182 is induced by IL-2 and promotes clonal expansion of activated helper T lymphocytes. Nat. Immunol. 2010; 11: 1057–1062. 10.1038/ni.1945 20935646

[5] Steiner DF, Thomas MF, Hu JK, Yang Z, Babiarz JE, Allen CDC, Matloubian M, et al MicroRNA-29 regulates T-box transcription factors and interferon-γ production in helper T cells. Immunity. 2011; 35: 169–181. 10.1016/j.immuni.2011.07.009 21820330 PMC3361370

[6] Ma F, Xu S, Liu X, Zhang Q, Xu X, Liu M, Hua M, et al The microRNA miR-29 controls innate and adaptive immune responses to intracellular bacterial infection by targeting interferon-γ. Nat. Immunol. 2011; 12: 861–869. 10.1038/ni.2073 21785411

[7] Liston A, Papadopoulou AS, Danso-Abeam D, Dooley J. MicroRNA-29 in the adaptive immune system: setting the threshold. Cell. Mol. Life Sci. 2012; 69: 3533–3541. 10.1007/s00018-012-1124-0 22971773 PMC11114856

[8] Lu L-F, Thai T-H, Calado DP, Chaudhry A, Kubo M, Tanaka K, Loeb GB, et al Foxp3-dependent microRNA155 confers competitive fitness to regulatory T cells by targeting SOCS1 protein. Immunity. 2009; 30: 80–91. 10.1016/j.immuni.2008.11.010 19144316 PMC2654249

[9] Du C, Liu C, Kang J, Zhao G, Ye Z, Huang S, Li Z, et al MicroRNA miR-326 regulates TH-17 differentiation and is associated with the pathogenesis of multiple sclerosis. Nat. Immunol. 2009; 10: 1252–1259. 10.1038/ni.1798 19838199

[10] Xue F, Li H, Zhang J, Lu J, Xia Y, Xia Q. miR-31 regulates interleukin 2 and kinase suppressor of ras 2 during T cell activation. Genes Immun. 2013; 14: 1–5. 10.1038/gene.2012.54 23303246

[11] Baumjohann D, Kageyama R, Clingan JM, Morar MM, Patel S, de Kouchkovsky D, Bannard O, et al The microRNA cluster miR-17~92 promotes TFH cell differentiation and represses subset-inappropriate gene expression. Nat. Immunol. 2013; 14: 840–848. 10.1038/ni.2642 23812098 PMC3720769

[12] Kang SG, Liu W-H, Lu P, Jin HY, Lim HW, Shepherd J, Fremgen D, et al MicroRNAs of the miR-17~92 family are critical regulators of T(FH) differentiation. Nat. Immunol. 2013; 14: 849–857. 10.1038/ni.2648 23812097 PMC3740954

[13] Mendell JT. miRiad roles for the miR-17-92 cluster in development and disease. Cell. 2008; 133: 217–222. 10.1016/j.cell.2008.04.001 18423194 PMC2732113

[14] Ventura A, Young AG, Winslow MM, Lintault L, Meissner A, Erkeland SJ, Newman J, et al Targeted deletion reveals essential and overlapping functions of the miR-17 through 92 family of miRNA clusters. Cell. 2008; 132: 875–886. 10.1016/j.cell.2008.02.019 18329372 PMC2323338

[15] Xiao C, Srinivasan L, Calado DP, Patterson HC, Zhang B, Wang J, Henderson JM, et al Lymphoproliferative disease and autoimmunity in mice with increased miR-17-92 expression in lymphocytes. Nat. Immunol. 2008; 9: 405–414. 10.1038/ni1575 18327259 PMC2533767

[16] Sylvestre Y, De Guire V, Querido E, Mukhopadhyay UK, Bourdeau V, Major F, Ferbeyre G, et al An E2F/miR-20a autoregulatory feedback loop. J. Biol. Chem. 2007; 282: 2135–2143. 17135249 10.1074/jbc.M608939200

[17] O’Donnell K A, Wentzel E A, Zeller KI, Dang C V, Mendell JT. c-Myc-regulated microRNAs modulate E2F1 expression. Nature. 2005; 435: 839–43. 15944709 10.1038/nature03677

[18] Wang Q, Li YC, Wang J, Kong J, Qi Y, Quigg RJ, Li X. miR-17-92 cluster accelerates adipocyte tumor-suppressor Rb2 / p130. Proc. Natl. Acad. Sci. 2008; 105: 2889–2894. 10.1073/pnas.0800178105 18287052 PMC2268555

[19] Jiang S, Li C, Olive V, Lykken E, Feng F, Sevilla J, Wan Y, et al Molecular dissection of the miR-17-92 cluster’s critical dual roles in promoting Th1 responses and preventing inducible Treg differentiation. Blood. 2011; 118: 5487–5497. 10.1182/blood-2011-05-355644 21972292 PMC3217351

[20] Cox MB, Cairns MJ, Gandhi KS, Carroll AP, Moscovis S, Stewart GJ, Broadley S, et al MicroRNAs miR-17 and miR-20a inhibit T cell activation genes and are under-expressed in MS whole blood. PLoS One. 2010; 5: e12132 10.1371/journal.pone.0012132 20711463 PMC2920328

[21] Arndt B, Poltorak M, Kowtharapu BS, Reichardt P, Philipsen L, Lindquist JA, Schraven B, et al Analysis of TCR activation kinetics in primary human T cells upon focal or soluble stimulation. J. Immunol. Methods. 2013; 387: 276–83. 10.1016/j.jim.2012.11.006 23178863

[22] Poltorak M, Arndt B, Kowtharapu BS, Reddycherla A V, Witte V, Lindquist JA, Schraven B, et al TCR activation kinetics and feedback regulation in primary human T cells. Cell Commun. Signal. 2013; 11: 4 10.1186/1478-811X-11-4 23317458 PMC3842781

[23] Nunes J, Bagnasco M, Lopez M, Lipcey C, Mawas C, Olive D. Dissociation between early and late events in T cell activation mediated through CD28 surface molecule. Mol Immunol. 1991; 28: 427–435 1648171 10.1016/0161-5890(91)90156-e

[24] Graves P, Zeng Y. Biogenesis of mammalian microRNAs: a global view. Genomics. Proteomics Bioinformatics. 2012; 10:, 239–45. 10.1016/j.gpb.2012.06.004 23200133 PMC5054211

[25] Smith-Garvin JE, Koretzky GA, Jordan MS. T cell activation. Annu. Rev. Immunol. 2009; 27: 591–619. 10.1146/annurev.immunol.021908.132706 19132916 PMC2740335

[26] Cebrián M, Yagüe E, Rincón M, López-Botet M, de Landázuri MO, Sanchez-Madrid F. Triggering of T cell proliferation through AIM, an activation inducer molecule expressed on activated human lymphocytes. J. Exp. Med. 1998; 28: 1621–1637. 10.1084/jem.168.5.1621 PMC2189112 2903209

[27] López-Cabrera M, Santis AG, Fernández-Ruiz E, Blacher R, Esch F, Sánchez-Mateos P, Sánchez-Madrid F. Molecular Cloning, expression and chromosomal localization of the human earliset lymphocyte activation antigen AIM/CD69, a new member of the C-type animal lectinin super family of signaling-transmitting receptors. J. Exp. Med. 1993; 178: 537–547. 8340758 10.1084/jem.178.2.537 PMC2191117

[28] Xiao C, Rajewsky K. MicroRNA control in the immune system: basic principles. Cell. 2009; 136: 26–36. 10.1016/j.cell.2008.12.027 19135886

[29] Van Haaften G, Agami R. Tumorigenicity of the miR-17-92 cluster distilled. Genes Dev. 2010; 24: 1–4. 10.1101/gad.1887110 20047995 PMC2802185

[30] Skinner JPJ, Keown AA, Chong MMW. The miR-17~92a Cluster of MicroRNAs Is Required for the Fitness of Foxp3(+) Regulatory T Cells. PLoS One. 2014; 9: e88997 10.1371/journal.pone.0088997 24523948 PMC3921252

[31] Wu T, Wieland A, Araki K. Temporal expression of microRNA cluster miR-17-92 regulates effector and memory CD8+ T-cell differentiation. Proc. Natl. Acad. Sci. 2012; 109: 9965–9970. 10.1073/pnas.1207327109 22665768 PMC3382487

[32] Liu SQ, Jiang S, Li C, Zhang B, Li QJ. miR-17-92 cluster targets phosphatase and tensin homology and Ikaros Family Zinc Finger 4 to promote TH17-mediated inflammation. J.Biol. Chem. 2014; 289: 12446–12456. 10.1074/jbc.M114.550723 24644282 PMC4007439

[33] Zhu D, Pan C, Li L, Bian Z, Lv Z, Shi L, Zhang J, Li D, Gu H, Zhang CY, Liu Y, Zen K. MicroRNA-17/20a/106a modulate macrophage inflammatory responses through targeting signal-regulatory protein α. J. Allergy Clin. Immunol. 2013; 132: 426–436. 10.1016/j.jaci.2013.02.005 23562609 PMC5882493

[34] Trompeter HI, Abbad H, Iwaniuk KM, Hafner M, Renwick N, Tuschl T, Schira J, Müller HW, Wernet P. MicroRNAs MiR-17, MiR-20a, and MiR-106b act in concert to modulate E2F activity on cell cycle arrest during neuronal lineage differentiation of USSC. PLoS One. 2011; 6: e16138 10.1371/journal.pone.0016138 21283765 PMC3024412

[35] Zhang M, Liu Q, Mi S, Liang X, Zhang Z, Su X, Liu J, Chen Y, Wang M, Zhang Y, Guo F, Zhang Z, Yang R. Both miR-17-5p and miR-20a alleviate suppressive potential of myeloid-derived suppressor cells by modulating STAT3 expression. J. Immunol. 2011; 186: 4716–4724. 10.4049/jimmunol.1002989 21383238

[36] Fontana L, Pelosi E, Greco P, Racanicchi S, Testa U, Liuzzi F, Croce CM, Brunetti E, Grignani F, Peschle C. MicroRNAs 17-5p-20a-106a control monocytopoiesis through AML1 targeting and M-CSF receptor upregulation. Nat. Cell Biol. 2007; 9: 775–787. 17589498 10.1038/ncb1613

[37] Milner JD, Fazilleau N, McHeyzer-Williams M, Paul W. Lack of high affinity competition for peptide in polyclonal CD4+ responses unmasks IL-4 production. J. Immunol. 2010; 184:6569–6573. 10.4049/jimmunol.1000674 20495070 PMC2930602

[38] Yamane H, Paul WE. Early signaling events that underlie fate decisions of naive CD4(+) T cells toward distinct T-helper cell subsets. Immunol Rev. 2013; 252: 12–23. 10.1111/imr.12032 23405892 PMC3578301

[39] van Panhuys N, Klauschen F, Germain RN. T-cell-receptor-dependent signal intensity dominantly controls CD4(+) T cell polarization In Vivo. Immunity. 2014; 41: 63–74. 10.1016/j.immuni.2014.06.003 24981853 PMC4114069

[40] Ebisuya M, Kondoh K, Nishida E. The duration, magnitude and compartmentalization of ERK MAP kinase activity: mechanisms for providing signaling specificity. J. Cell. Sci. 2005; 118: 2997–3002. 16014377 10.1242/jcs.02505

[41] Amil CF, Oppenheim JJ, Zachariae COC, Mukaida N, Matsushima K. Properties of the novel proinflammatory supergene ''Intercrine'' cytokine family ^1^ Annu.Rev. Immunol. 1991; 9: 617–648. 1910690 10.1146/annurev.iy.09.040191.003153

[42] Dennis D. Taub, Miriam Anver, Joost J. Oppenheim, Dan L. Longo and William J. Murphy. T Lymphocyte Recruitment by Interleukin-8 (IL-8). J. Clin. Invest. 1996; 97: 1931–1941. 8621778 10.1172/JCI118625 PMC507263

[43] Yu Z, Willmarth NE, Zhou J, Katiyar S, Wang M, Liu Y, McCue PA, et al microRNA 17/20 inhibits cellular invasion and tumor metastasis in breast cancer by heterotypic signaling. Proc. Natl. Acad. Sci. 2010; 107: 8231–8236. 10.1073/pnas.1002080107 20406904 PMC2889540

[44] Assier E, Boissier M-C, Dayer J-M. Interleukin-6: from identification of the cytokine to development of targeted treatments. Joint. Bone. Spine. 2010; 77: 532–536. 10.1016/j.jbspin.2010.07.007 20869898

[45] Tanaka T, Narazaki M, Ogata A, Kishimoto T . A new era for the treatment of inflammatory autoimmune diseases by interleukin-6 blockade strategy. Semin. Immunol. 2014; 26: 88–96. 10.1016/j.smim.2014.01.009 24594001

[46] Rincon M. Interleukin-6: from an inflammatory marker to a target for inflammatory diseases. Trends Immunol. 2012; 33: 571–577. 10.1016/j.it.2012.07.003 22883707

[47] Sabat R, Grütz G, Warszawska K, Kirsch S, Witte E, Wolk K, Geginat J. Biology of interleukin-10. Cytokine Growth Factor Rev. 2010; 21: 331–344. 10.1016/j.cytogfr.2010.09.002 21115385

[48] Ng THS, Britton GJ, Hill E V, Verhagen J, Burton BR, Wraith DC. Regulation of adaptive immunity; the role of interleukin-10. Front. Immunol. 2013; 4: 1–13. 10.3389/fimmu.2013.00001 23755052 PMC3668291

[49] Hutchins AP, Diez D, Miranda-Saavedra D. The IL-10/STAT3-mediated anti-inflammatory response: recent developments and future challenges. Brief. Funct. Genomics. 2013; 12: 489–498. 10.1093/bfgp/elt028 23943603 PMC3838198

[50] Dumont FJ, Staruch MJ, Fischer P, DaSilva C, Camacho R. Inhibition of T cell activation by pharmacologic disruption of the MEK1/ERK MAP kinase or calcineurin signaling pathways results in differential modulation of cytokine production. J. Immunol. 1998; 160: 2579–2589. 9510155

[51] Saraiva M, Christensen JR, Veldhoen M, Murphy TL, Murphy KM, O’Garra A. Interleukin-10 production by Th1 cells requires interleukin-12-induced STAT4 transcription factor and ERK MAP kinase activation by high antigen dose. Immunity. 2009; 31: 209–219. 10.1016/j.immuni.2009.05.012 19646904 PMC2791889

[52] Dodeller F, Skapenko A, Kalden JR, Lipsky PE, Schulze-Koops H. The p38 mitogen-activated protein kinase regulates effector functions of primary human CD4 T cells. Eur. J. Immunol. 2005; 35: 3631–42. 16259005 10.1002/eji.200535029

[53] Sloan DD1, Jerome KR. Herpes simplex virus remodels T-cell receptor signaling, resulting in p38-dependent selective synthesis of interleukin-10. J. Virol. 2007; 81: 12504–14. 17804501 10.1128/JVI.01111-07 PMC2169026

[54] Buckler JL, Liu X, Turka LA. Regulation of T-cell responses by PTEN. Immunol. Rev. 2008; 224: 239–248. 10.1111/j.1600-065X.2008.00650.x 18759931 PMC2876726

[55] Zhang M, Liu Q, Mi S, Liang X, Zhang Z, Su X, Liu J, et al Both miR-17-5p and miR-20a alleviate suppressive potential of myeloid-derived suppressor cells by modulating STAT3 expression. J. Immunol. 2011; 186: 4716–4724. 10.4049/jimmunol.1002989 21383238

[56] Psathas JN, Doonan PJ, Raman P, Freedman BD, Minn AJ, Thomas-Tikhonenko A. The Myc-miR-17-92 axis amplifies B-cell receptor signaling via inhibition of ITIM proteins: a novel lymphomagenic feed-forward loop. Blood. 2013; 122: 4220–9. 10.1182/blood-2012-12-473090 24169826 PMC3868926

